# 

**Published:** 1909-12-15

**Authors:** 


CONTENTS

Event and Comment - - - - - - - - 605

A Comparison of Old and New Style Attachments in Fixed Bridges, *By R. B. Howell, D.D.S.* 608

Dental Fees, - - - - - - *By Brother Bill* 620

Some Practical Points on Taggart Inlays *By L. E. Custer, A.M., D.D.S.* 627

Paraform in the Treatment of Sensitive Dentine *By J. Ainsworth Woods, L.D.S.* 630

In Memoriam - - - - - - - - - - 633

Indents - - - - - - - - - - 634

Announcements - - - - - - - - - 648

